# Serum Lipid Levels and Suicidal Ideation of Adults: A Cross-Sectional Study Using the Korea National Health and Nutrition Examination Survey

**DOI:** 10.3390/jcm12134285

**Published:** 2023-06-26

**Authors:** Hana Cho, Jinyoung Shin, Jae Kyung Choi

**Affiliations:** Department of Family Medicine, Konkuk University Medical Center, Konkuk University School of Medicine, Seoul 05030, Republic of Korea; 20210100@kuh.ac.kr (H.C.); cjk@kuh.ac.kr (J.K.C.)

**Keywords:** low-density lipoprotein, household income, education level, suicidality, sex

## Abstract

Cholesterol plays a crucial role in the brain, which suggests that changes in its concentration levels may have an impact on the central nervous system. To examine the association between serum lipid levels and suicidal ideation according to sex, we performed a cross-sectional study using data from the Korea National Health and Nutrition Examination Survey 2014–2018. A total of 13,772 adults 19 years or older were analyzed. The ninth item of the Patient Health Questionnaire was used to evaluate the suicidal ideation of participants. After sorting by sex, a complex logistic regression was performed to measure the association between serum lipid indicators and suicidal ideation. The analysis adjusted for age, body mass index, smoking, heavy drinking, regular exercise, household income, education level, dyslipidemia medication, depression, and chronic diseases. Compared to the intermediated category, the lowest range of low-density lipoprotein cholesterol (LDL-C; <100 mg/dL) was associated with increased suicidal ideation in men (odds ratio [OR] = 1.97; 95% confidence interval [CI]: 1.30–3.01). The association between lipid levels and suicidal ideation was not clear in women. We found an association between lower LDL-C levels and an increased risk of suicidal ideation among Korean men aged 19 years or older.

## 1. Introduction

Suicide is a serious health problem in South Korea. In 2013, South Korea’s suicide rate was 2.4 times higher than the average suicide rate of other Organization for Economic Co-operation and Development (OECD) countries (28.5 per 100,000 person-years), and South Korea has continued to rank top among OECD countries in suicide rates for the past 10 years [1]. The causes of suicide attempts vary widely depending on geographic regions, genetics, external risk factor profiles, and neurobiology [2]. One study by Kim et al. found depression and poor social support to be statistically significant risk factors for suicidal ideation among older adults in South Korea [3]. The study also identified patients with depression to have significantly reduced total serum cholesterol levels compared to the control group when matched by gender, age, and weight. Furthermore, the patients with depression who attempted suicide showed a significant decrease in total serum cholesterol levels compared to patients with depression who did not attempt suicide [4].

Cholesterol is vital for cell membrane stability and the proper functioning of neurotransmissions. It also plays a crucial role in the second messenger system of the brain, which is associated with the mechanics of antidepressant drugs and mood stabilizers [5,6]. Logic then follows that changes in cholesterol concentrations in the brain may have an impact on the central nervous system.

There have been many studies examining the connection between cholesterol levels and suicidal behavior. These studies have typically used cholesterol level as a biomarker to reflect the risk of suicidality. A cholesterol level of less than 4.14 mmol/L (<160 mg/dL) has been associated with an increased risk of death from suicide [7]. A seven-year prospective study found that total cholesterol (TC) and low-density lipoprotein cholesterol (LDL-C) values were lower in men who reported low mood, depression, or anxiety. The same study found that men with low cholesterol concentrations had 4.2 times higher suicide attempts than men with the highest cholesterol levels [8]. Another study conducted on the Chinese population found that suicidal patients had meaningfully lower TC, high-density lipoprotein cholesterol (HDL-C), and LDL-C levels compared to healthy controls [9]. 

Other studies examined the cholesterol levels in psychiatric patients admitted to an emergency ward following an attempted suicide. These studies found that TC concentrations in suicide attempters were significantly lower compared to both psychiatric and normal controls [10]. The meta-analysis of major depressive disorder (MDD) patients found that lower concentrations of TC and LDL-C, but not of HDL-C and Triglycerides (TG), were associated with attempted suicide [11]. 

In conflict with studies that showed an association between low serum cholesterol levels and increased suicidal risk [12,13], some researchers have reported no direct correlation between low cholesterol levels and mortality from impulsive suicide [14,15]. Studies on older Korean populations found that lower TC levels correlated with a reduced risk of suicidal ideation, while HDL-C, LDL-C, and TG were irrelevant to suicidal ideation [16]. Shaker et al. found a statistically significant connection between higher LDL and negative self-image [17]. Pompili M. et al. reported that among mood disorder patients, suicidal attempters and non-attempters did not differ in the levels of serum cholesterol or TG [18]. Furthermore, Park et al. reported no association between suicide and the levels of serum lipid metabolites in psychiatric inpatients with schizophrenia, bipolar affective disorder, or MDD [19]. As such, the link between serum cholesterol levels and suicidality remains inconsistent. 

In this study, we examined the association between serum lipid levels and suicidal ideation according to sex, using a representative sample of Korean adults.

## 2. Materials and Methods

### 2.1. Study Participants

The study used a nationally representative dataset from the 2014, 2016, and 2018 sixth and seventh Korea National Health and Nutrition Examination Survey (KNHNES). KNHNES collects information on socioeconomic status, health-related behaviors, quality of life, healthcare utilization, anthropometric measures, biochemical and clinical profiles for non-communicable diseases, and dietary intake via a health interview, health examination, and a nutrition survey [20]. 

**Figure 1:**
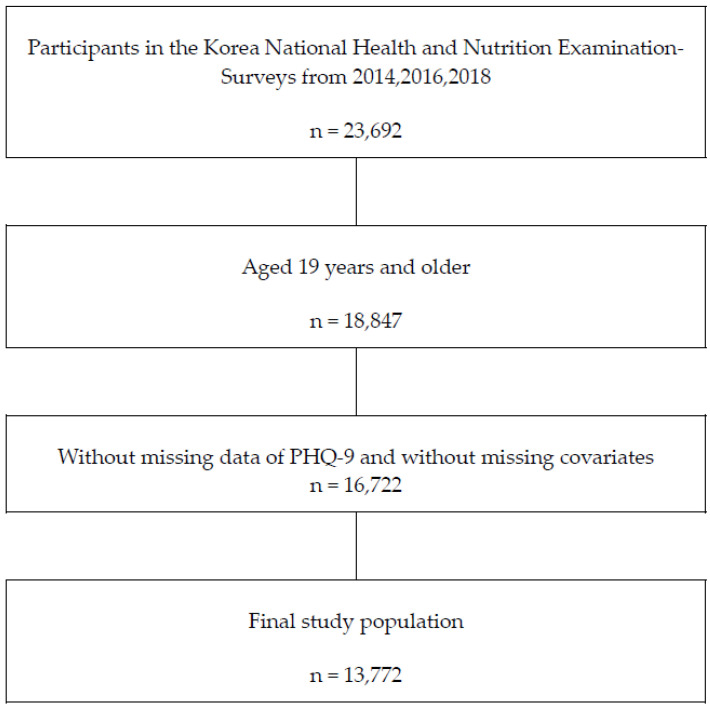
Flow diagram of the participants. PHQ-9 Patient Health Queistionnaire-9.

This study is a secondary analysis of data from the KNHNES with a cross-sectional study design. Of the 18,847 participants aged 19 years or older, 2125 did not complete the Patient Health Questionnaire (PHQ) and were excluded. Among the remaining participants, those with missing data in at least one of the covariates and those who did not provide an answer on suicidality and blood samples were also excluded. A dataset of the remaining 13,772 participants was analyzed for this study (Figure 1). 

### 2.2. Measurement of Lipid Levels

LDL-C, HDL-C, TC, and TG serum levels were measured and collected from the blood samples of individual participants who fasted for at least 8 h. KNHNES measured HDL-C levels based on the Lipid Standardization Program by the Centers for Disease Control (CDC) [21]. TC, TG, LDL-C, and HDL-C were measured using a Hitachi Automatic Analyzer 7600 (Hitachi, Tokyo, Japan). The Friedewald equation was used to calculate LDL-C levels in TG levels below 200 mg/dL [22]. In TG levels above 200 mg/d, the direct measurement of LDL-C levels was used.

### 2.3. Assessment of Suicidal Ideation

The PHQ is a self-reported questionnaire designed to help detect and diagnose mental disorders common in primary clinical settings [23]. To evaluate the suicidality of the participants, we used the ninth item of the PHQ-9, which asked, “Have you felt that you would be better off dead or of hurting yourself over the past 2 weeks?” If the ninth item of the PHQ-9 had a score of ≥1, including “several days”, “more than half the days”, or “nearly every day”, the participant was categorized as suicidal. If the ninth item of the PHQ-9 was 0 or “not at all”, the participant was categorized as not suicidal.

### 2.4. Covariates

The variables measured were age, height, body mass index (BMI), household income, education, depression, current smoking status, heavy drinking, regular exercise, dyslipidemia medication, and chronic disease. Household income was divided into four quartiles from the lowest to the highest. Education level was sorted into four categories: elementary school or lower; middle school; high school; and college or higher. Depression was defined as having depression diagnosed by a physician. Based on their smoking status, subjects were categorized into three groups: never-smokers; ex-smokers; and current smokers. Heavy drinking was defined as “seven servings or more” at a time for males and “five servings or more” at a time for females, at least twice a week. Regular exercise was divided into “at least once per week” or not. The current use of lipid-lowering medication was confirmed using a self-administered survey. Having a chronic disease was defined as being previously diagnosed with a stroke, myocardial infarction or angina, diabetes mellitus, chronic kidney disease, any malignancy, liver cirrhosis, rheumatoid arthritis, osteoarthritis, or asthma.

### 2.5. Statistical Analysis

All statistics in this survey were calculated using sample weights, which were designed for the sample participants representative of the Korean population. The weights were based on the inverse of selection probabilities, and the inverse of response rates was modified by adjusting them to the sex-and age-specific Korean populations [20].

After sorting according to sex, an independent *t*-test was used for the continuous variables. For the categorical variables, a chi-square test was performed to determine the differences in variables. 

To determine the association between serum lipid indicators and suicidality, a complex logistic regression analysis was performed. Model 1 was executed without covariates, using univariate logistics regression. Model 2 was obtained after adjusting for age (categorical), BMI (categorical), smoking status, heavy drinking, and regular exercise. Model 3 was obtained after making an additional adjustment for socioeconomic variables, household income, and education level. Model 4 was further adjusted for dyslipidemia medication, depression, and chronic disease. Categories of serum lipid levels were set as follows: LDL-C < 100 mg/dL; LDL-C 100–129 mg/dL; LDL-C 130–159 mg/dL; LDL-C 160–189 mg/dL; LDL-C ≥ 190 mg/dL; TC < 200 mg/dL; TC 200–239 mg/dL; TC ≥ 240 mg/dL; HDL-C < 40 mg/dL; HDL-C 40–59 mg/dL; HDL-C > −60 mg/dL; TG < 150 mg/dL; TG 150–199 mg/dL; and TG ≥ 200 mg/dL. A logistic regression analysis was carried out to calculate odd ratios (OR) and 95% confidence intervals to identify the risk of low lipid levels according to suicidal ideation. Furthermore, we performed a stratified analysis to examine how these associations differed by household income and education level, using complex logistic regression analysis. Statistical analysis was performed using IBM SPSS version 27.0 (SPSS Inc, Chicago, IL, USA), and the statistical significance was set to *p* < 0.05.

## 3. Results

### 3.1. Characteristics of Participants

Of the 13,772 total participants analyzed, 5721 (41.5%) were men, and 8051 (58.5%) were women. The characteristics of the participants are shown in Table 1. Among men, 268 (4.7%) had suicidal ideation, whereas 606 (7.5%) women had suicidal ideation.

The independent *t*-test and chi-square test showed significant differences in age (categorical), household income, education level, depression, chronic diseases, and LDL-C and TC levels between men with and without suicidal ideation. Between women with and without suicidal ideation, there were significant differences in age (categorical), height, household income, education level, depression, smoking status, heavy drinking, regular exercise, dyslipidemia medication, and HDL-C and TG levels. Of participants aged 75 and older, the group diagnosed with depression and chronic diseases and the group with the lowest income had higher rates of suicidal ideation.

### 3.2. Relationship between Lipid Levels and Suicidality in Men and Women

The lowest range of LDL-C was related to increased suicidal ideation in men (Table 2). Men showed an inverse association between TC level and suicidal ideation, although the association was insignificant once adjusted for socioeconomic variables. For TG, the positive association with suicidal ideation was withdrawn after adjusting for dyslipidemia medication, chronic diseases, and depression. HDL-C did not reveal a statistically significant association with suicidal ideation. 

In women, an inconsistent association was seen in the lowest TC, HDL-C, and the highest TG levels and then was attenuated after the covariate adjustment (Table 3). 

### 3.3. Relationship between Household Income, Education Levels, and Suicidality 

In both men and women, suicidal ideation was higher in the lowest household income compared to the fourth quartile household income (Supplementary Table S1). In men, there was a statistically significant relationship between the lowest household income and suicidal ideation (OR = 5.082; 95% CI = 3.283–7.865). In women, there was a higher probability of suicidal ideation in the lowest household income, second quartile income, and third quartile income, using the highest quartile household income as the reference category (OR = 4.962, 95% CI = 3.66–6.726; OR = 2.087, 95% CI = 1.509–2.887; OR = 1.57, 96% CI = 1.134–2.174). 

In both men and women, the number of participants with suicide ideation increased in the lower income and lower education levels. 

In men, LDL-C, TC, and HDL-C were positively associated with income and education levels, as seen in Supplementary Table S2. However, in women, only HDL-C was positively associated with income and education levels, while TG was reversely associated with income and education levels. 

## 4. Discussion

This study found that an LDL-C level lower than 100 mg/dL was significantly related to increased suicidal ideation in men, even after adjusting for covariates.

Past publications examining mostly TC levels have shown an association between cholesterol levels and suicidal behavior in adults with psychiatric illnesses. However, in more recent studies, the association between suicidality and LDL-C remains inconsistent [2]. The findings from this study support other studies that show an association between low LDL-C and suicidality. In addition, this study saw no association between TC and suicidality once adjusted for income and education levels, which indirectly confirms an association between lipid levels and income and education levels. Nevertheless, this study found that there is an independent association between low LDL-C and suicide ideation in Korean men. 

High-lethality suicide attempters had significantly lower LDL-C compared to low-lethality suicide attempters and inpatients who never attempted suicide [24]. Additionally, Ayesa-Arriola et al. showed that low levels of LDL-C and depressive symptoms were meaningfully associated with suicidal behavior in first-episode psychosis (FEP) patients, and more psychotic symptoms were related to lower LDL-C [25]. Several theories have been suggested to explain the positive association between low LDL-C and suicidal ideation. A possible neurobiological theory presented by Engelberg et al. [12] suggests that low serum cholesterol could cause a decrease in the number of brain serotonin receptors, which, in turn, could increase violent behavior. He hypothesized that a decreased serum cholesterol level could lead to a decrease in the viscosity of the neuronal membranes. This would then lead to a failure in synaptic transmission as well as the functioning of serotonin receptors and transporters. Such an event would cause a decrease in serotonin intake via serotonin receptors (5-HT1A), which may lead to an increase in suicide ideation [26]. In addition, a decrease in serum TC or LDL-C would induce a comparative increase in brain cell membrane fluidity with increased presynaptic serotonin reuptake and reduced postsynaptic serotonin function [27].

Fisher et al. postulated a two-way relationship between cholesterol and the serotonergic system with a connection between LDL-C levels and the alleles coding for serotonin transporter polymorphism (5HTTLPR) [28]. The study revealed that 5HTTLPR has an influence on LDL-C levels in women. The serotonin transporter gene’s long allele was associated with higher LDL-C, and the short allele with lower LDL-C. Therefore, gender differences in this study may be attributed to the different transporter polymorphisms. 

This study also found that high levels of TG have a positive correlation with suicidal ideation in men but not in the fully adjusted model. This trend can be explained by the association between the confounding variables, such as depression and high TG levels. In previous studies, a causal relationship was found between high TG and depressive symptoms in men [29,30]. Moreover, a recent Korean study showed that the strongest predictor of suicidal ideation was depression in men, which can be supported by the psycho-immunological theory [31]. In depressed patients, increased production of cytokines, such as interleukin-2 (IL-2) from the activation of T-cell-mediated immune reaction, lowers serum TC and HDL-C levels while increasing TG to suppress the secretion of melatonin from the pineal gland. This eventually leads to depression and suicidal behavior [32]. Among women, there was no consistently significant association between any cholesterol level and suicidal ideation. 

As for the socioeconomic indicators, the results of this study showed gender differences in the relationship between household income, education levels, and lipid levels, such as LDL-C and TC. Male participants with the lowest household income and education levels were more likely to have the lowest LDL-C, TC, and HDL-C levels (Supplementary Table S2). In women, HDL-C showed the same trend as in men, and women with high incomes had lower levels of TG. There was a similar trend for the most educated women. In women with lower incomes, HDL-C showed the same trend as in men, and women with high incomes had lower levels of TG. There was a similar trend for the most educated women (Supplementary Table S3). The more privileged men had higher LDL-C, HDL-C, and TC, whereas a higher ratio of women with the highest education level had low LDL-C, TC, TG, and high HDL-C. 

Zhan et al. reported that the prevalence of high TG and low HDL-C decreased with higher educational levels among Chinese women. The gender differences may be explained by the special economic transition period. Chinese men with higher SES had much more ability and opportunity to consume fatty foods, which could have resulted in higher TG and lower HDL. In contrast, Chinese women tended to be more concerned about their fitness [33]. Therapy could be something to explore with this population. Liraglutide showed significant weight loss in overweight women by promoting insulin secretion and limiting glucagon according to glucose changes [34]. Obese women with lower education levels and lower personal incomes, yet with a higher level of triglycerides, can be a possible case of liraglutide. A differential in the relationship between socioeconomic indicators and serum lipids between men and women was shown in other studies in Korea [35,36]. Lower education level and lower personal income were associated with a higher risk of metabolic syndrome in Korean women but not in Korean men. Women of higher SES were more likely to be more concerned about their health and fitness [37]. Moreover, the differences in dietary choices, such as fruit and vegetable consumption, were likely contributors to health inequalities [38,39]. Educational attainment was a strong socioeconomic indicator of disparities in dietary intake due to the close relationship between nutritional knowledge and health consideration in food choices [40,41]. Similarly to China, rapid globalization and technological modernization have resulted in rapid changes in Korean society. For instance, the consumption of snacks and excessive cholesterol-rich foods and SES might be vital factor in the early phases of eating behavior transition in Korea [37]. 

The study had several limitations. Due to the study’s cross-sectional design, the changes in serum lipid levels were not considered, and, therefore, a causal relationship between LDL-C levels and suicidal ideation cannot be inferred. This study also did not consider several confounding variables, such as diet habits and other mental health history, with the exception of depression. Our study defined depression as the presence or absence of a history of depression diagnosis by a clinician. Depression is defined as a medical condition that lasts more than two consecutive weeks, including sadness, loss of interest, changes in appetite, sleeping problems, fatigue, purposeless physical activity or slowed movements, feeling worthless or guilty, difficulty thinking, and thoughts of suicide [42]. However, environmental, psychological, and genetic factors, which were not adjusted in this study, might all be involved in complex interactions, which could result in depression [43]. Moreover, suicidal ideation was only acquired by the self-administered ninth item of the PHQ-9 questionnaire, and the study did not evaluate the information regarding suicidal attempts or the completion of serious suicidal behavior.

Statins have a great potential to lower the risk of developing cardiovascular disease (CVD) by decreasing serum levels of LDL-C. Currently, more than 25 million individuals use statins worldwide [44]. In South Korea, the 2015 Korean guidelines for the management of dyslipidemia advised LDL-C goals based on an individual’s cardiovascular risk level, similar to the National Cholesterol Education Program (NCEP) Adult Treatment Panel III (ATP III) guidelines [45]. The 2015 Korean guidelines defined a very high-risk group as patients with established CVD and a recommended goal of LDL-C being less than 70 [46,47]. There may be many individuals whose LDL-C has been lowered to less than 100 by taking statins. Further studies would be needed to categorize LDL-C less than 100 with consideration for statin treatments.

Regardless of these limitations, the findings suggest an inverse association between serum LDL-C and suicidal ideation in men among the Korean adult population. In addition, suicidal thoughts showed an increasing trend in both low-level LDL and high-level LDL. Tomson-Johanson et al. studied the effects of LDL on impulsivity. They confirmed that high serum LDL levels were associated with increased impulsivity and decreased inhibition in adult girls [48,49]. High serum LDL levels could be related to psychological reasons, such as depression, suicide, motor, cognition, and unplanned impulsivity, in patients with morbid obesity [50]. Future longitudinal and large-sized studies would be needed to evaluate the causality of lipid profiles and suicidal ideation. This study should also be replicated in different cultures or geographical contexts to confirm the findings’ generalizability. 

## 5. Conclusions

This study showed that there is an independent association between lower LDL-C levels and increased risk of suicidal ideation among Korean men aged 19 years or older. Socioeconomic variables, such as household income and education levels, may influence that association.

## Figures and Tables

**Table 1:** Characteristics of study participants according to suicidal ideation.

Variables		Men			Women		
		*n* = 5721			*n* = 8051		
		Suicidal Ideation		*p*-Value	Suicidal Ideation		*p*-Value
		No	Yes		No	Yes	
		*n* = 5453	*n* = 268		*n* = 7445	*n* = 606	
Mean ± SD		45.43 ± 0.3	46.79 ± 1.32	0.305	46.86 ± 0.27	52.22 ± 0.93	<0.001
Age	19–39	1585 (39.23)	62 (37.9)	0.007	2160 (35.79)	118 (27.38)	<0.001
	40–74	3381 (56.04)	156 (52.43)		4676 (58.47)	392 (60.85)	
	≥75	487 (4.72)	50 (9.68)		609 (5.74)	96 (11.78)	
BMI	Normal (<23)	1826 (33.48)	114 (37.91)	0.211	3724 (53.4)	275 (49.07)	0.103
	Overweight (23–25)	1427 (25.55)	55 (20.2)		1557 (19.58)	126 (19.42)	
	Obese (≥25)	2200 (40.97)	99 (41.89)		2164 (27.02)	205 (31.51)	
Household Income	1 Quartile	840 (11.49)	113 (34.19)	<0.001	1312 (14.57)	253 (35.95)	<0.001
	2 Quartile	1334 (23.27)	59 (21.37)		1847 (24.48)	155 (25.42)	
	3 Quartile	1597 (31.26)	55 (24.53)		2082 (29.35)	126 (22.92)	
	4 Quartile	1682 (33.99)	41 (19.91)		2204 (31.6)	72 (15.72)	
Education	≤Elementary school	788 (8.94)	77 (18.37)	<0.001	1705 (16.95)	289 (38.87)	<0.001
level	Middle school	580 (7.93)	34 (9.14)		741 (8.97)	75 (11.74)	
	High school	1882 (37.96)	90 (41.6)		2401 (35.91)	132 (27.55)	
	≥University	2203 (45.18)	67 (30.88)		2598 (38.17)	110 (21.84)	
Depression	No	5353 (98.23)	234 (87.28)	<0.001	7059 (94.82)	459 (75.52)	<0.001
		100 (1.77)	34 (12.72)		386 (5.18)	147 (24.48)	
Smoking	Nonsmoker/ex-smoker	3582 (62.69)	165 (57.02)	0.112	7122 (94.92)	530 (85.19)	<0.001
	Current smoker	1871 (37.31)	103 (42.98)		323 (5.08)	76 (14.81)	
Heavy drinking	yes	995 (19.63)	60 (25.2)	0.082	371 (5.71)	44 (9.25)	0.009
Regular exercise (≥1 time/week)	yes	1785 (34.03)	64 (28.36)	0.128	1248 (17.68)	75 (12.34)	0.003
Dyslipidemia medication	yes	541 (7.77)	26 (7.37)	0.819	918 (9.96)	130 (17.2)	<0.001
Chronic disease	(One or more)	1261 (17.05)	99 (25.97)	<0.001	2106 (23.98)	302 (43.18)	<0.001
LDL cholesterol (mg/dL)	<100	1884 (33.92)	113 (44.92)	0.004	2456 (33.45)	209 (35.8)	0.400
	100–129	1897 (35.05)	90 (33.81)		2761 (38.09)	218 (38.25)	
	130–159	1260 (23.23)	44 (13.87)		1570 (20.25)	119 (16.87)	
	160–189	350 (6.66)	17 (6.17)		542 (6.64)	46 (7.04)	
	≥190	62 (1.14)	4 (1.23)		116 (1.57)	14 (2.04)	
Total cholesterol (mg/dL)	<200	3420 (61.89)	176 (69.34)	0.066	4548 (62.27)	370 (64.98)	0.288
	200–239	1576 (29.39)	66 (22.66)		2176 (28.54)	172 (25.16)	
	≥240	457 (8.73)	26 (8)		721 (9.19)	64 (9.86)	
HDL cholesterol (mg/dL)	<40	1408 (24.36)	77 (27.37)	0.471	788 (9.41)	89 (13.42)	0.006
	40–59	3351 (62.93)	161 (62.29)		4389 (57.83)	366 (59.76)	
	≥60	694 (12.7)	30 (10.34)		2268 (32.75)	151 (26.82)	
Triglyceride (mg/dL)	<150	3335 (60.67)	144 (52.07)	0.067	5866 (80.59)	425 (71.74)	<0.001
	150–199	919 (16.82)	52 (19.15)		842 (10.17)	87 (13.64)	
	≥200	1199 (22.51)	72 (28.77)		737 (9.24)	94 (14.62)	

**Table 2:** Association between suicidal ideation and lipid levels in men.

		Men							
		Model 1		Model 2		Model 3		Model 4	
		OR (95% CI)	*p*-Value	OR (95% CI)	*p*-Value	OR (95% CI)	*p*-Value	OR (95% CI)	*p*-Value
LDL Cholesterol (mg/dL)	<100	2.218 (1.457, 3.375)	<0.001	2.109 (1.378, 3.229)	0.001	1.905 (1.255, 2.891)	0.003	1.979 (1.300, 3.014)	0.002
	100–129	1.615 (1.021, 2.557)	0.041	1.593 (1, 2.538)	0.05	1.504 (0.947, 2.388)	0.084	1.568 (0.987, 2.491)	0.057
	130–159	1		1		1		1	
	160–189	1.552 (0.808, 2.981)	0.187	1.509 (0.785, 2.900)	0.217	1.454 (0.755, 2.803)	0.263	1.412 (0.714, 2.793)	0.320
	≥190	1.818 (0.600, 5.507)	0.290	1.806 (0.591, 5.519)	0.299	1.777 (0.561, 5.627)	0.327	1.876 (0.597, 5.898)	0.281
Total Cholesterol (mg/dL)	<200	1.453 (1.042, 2.027)	0.028	1.464 (1.048, 2.046)	0.026	1.379 (0.986, 1.930)	0.06	1.401 (1.000, 1.962)	0.05
	200–239	1		1		1		1	
	≥240	1.189 (0.722, 1.956)	0.496	1.170 (0.704, 1.944)	0.544	1.199 (0.713, 2.016)	0.493	1.112 (0.646, 1.913)	0.701
HDL Cholesterol (mg/dL)	<40	1.380 (0.832, 2.290)	0.212	1.470 (0.844, 2.56)	0.173	1.320 (0.747, 2.332)	0.339	1.293 (0.741, 2.256)	0.365
	40–59	1.216 (0.758, 1.952)	0.417	1.276 (0.769, 2.115)	0.345	1.211 (0.721, 2.034)	0.469	1.215 (0.734, 2.009)	0.448
	≥60	1		1		1		1	
Triglyceride (mg/dL)	<150	1		1		1		1	
	150–199	1.327 (0.898, 1.962)	0.156	1.325 (0.883, 1.988)	0.174	1.316 (0.872, 1.984)	0.191	1.336 (0.886, 2.016)	0.166
	≥200	1.489 (1.016, 2.181)	0.041	1.486 (1.003, 2.201)	0.048	1.503 (1.007, 2.244)	0.046	1.454 (0.976, 2.165)	0.066

Model 1: no adjustments; Model 2: adjusted for age, BMI, smoking status, heavy drinking, and regular exercise; Model 3: Model 2 + adjusted for household income and education level; Model 4: Model 3 + adjusted for dyslipidemia medication, depression, and chronic disease.

**Table 3:** Association between suicidal ideation and lipid levels in women.

						Women			
		Model 1		Model 2		Model 3		Model 4	
		OR (95% CI)	*p*-Value	OR (95% CI)	*p*-Value	OR (95% CI)	*p*-Value	OR (95% CI)	*p*-Value
LDL cholesterol	<100	1.284 (0.982, 1.679)	0.068	1.315 (0.998, 1.734)	0.052	1.296 (0.981, 1.712)	0.068	1.24 (0.919, 1.673)	0.158
	100–129	1.205 (0.921, 1.575)	0.173	1.26 (0.963, 1.647)	0.091	1.313 (1.001, 1.722)	0.049	1.326 (1, 1.759)	0.050
	130–159	1		1		1		1	
	160–189	1.272 (0.842, 1.922)	0.252	1.187 (0.775, 1.818)	0.431	1.158 (0.759, 1.766)	0.495	1.142 (0.738, 1.768)	0.549
	≥190	1.564 (0.808, 3.026)	0.184	1.446 (0.758, 2.76)	0.263	1.357 (0.728, 2.53)	0.336	1.484 (0.78, 2.825)	0.228
Total cholesterol	<200	1.184 (0.949, 1.476)	0.134	1.256 (1.001, 1.577)	0.049	1.26 (0.997, 1.593)	0.053	1.259 (0.983, 1.613)	0.068
	200–239	1		1		1		1	
	≥240	1.217 (0.856, 1.731)	0.273	1.172 (0.821, 1.674)	0.381	1.138 (0.793, 1.632)	0.482	1.157 (0.796, 1.683)	0.445
HDL cholesterol	<40	1.741 (1.229, 2.464)	0.002	1.413 (0.996, 2.004)	0.053	1.202 (0.841, 1.719)	0.312	1.108 (0.77, 1.596)	0.579
	40–59	1.262 (0.996, 1.599)	0.054	1.198 (0.942, 1.523)	0.140	1.117 (0.872, 1.431)	0.379	1.103 (0.857, 1.419)	0.447
	≥60	1		1		1		1	
Triglyceride	<150	1		1		1		1	
	150–199	1.507 (1.116, 2.035)	0.008	1.321 (0.972, 1.796)	0.075	1.216 (0.895, 1.652)	0.211	1.15 (0.843, 1.567)	0.377
	≥200	1.777 (1.355, 2.33)	<0.001	1.499 (1.129, 1.991)	0.005	1.337 (0.998, 1.791)	0.051	1.307 (0.97, 1.762)	0.078

Model 1: no adjustments; Model 2: adjusted for age, BMI, smoking status, heavy drinking, and regular exercise; Model 3: Model 2 + adjusted for household income and education level; Model 4: Model 3 + adjusted for dyslipidemia medication, depression, and chronic disease.

## Data Availability

The data presented in this study are available on request from the corresponding author.

## Supplementary Materials

The following are available online at https://www.mdpi.com/article/10.3390/jcm12134285/s1, Table S1: The risk of suicidal ideation according to the Household income and Education levels. Table S2: Relationship between lipid levels and household income & education levels in Men. Table S3: Relationship between lipid levels and household income & education levels in Women.
