# Supplementary material for: Serum Lipid Levels and Suicidal Ideation of Adults: A Cross-Sectional Study Using the Korea National Health and Nutrition Examination Survey
Source: J Clin Med. 2023 Jun 26;12(13):4285. doi: 10.3390/jcm12134285 (PMC10342697; doi:10.3390/jcm12134285)
Supplement: Supplementary file 1 [file jcm-12-04285-s001.zip › jcm-2418836-supplementary.pdf]

**Supplementary Table S1. The risk of suicidal ideation according to the Household income and Education levels**

| | | Men | | | Women | | |
|---|---|---|---|---|---|---|---|
| | | *n*=5,721 | | | *n*=8,051 | | |
| | | PHQ9 (Suicidal ideation) – 9th | | *p*-Value | PHQ9 (Suicidal ideation) – 9th | | *p*-Value |
| | | No<br>n=5,453 | Yes<br>n=268 | | No<br>n=7,445 | Yes<br>n=606 | |
| Household Income | 1 Quartile | 840(87.9) | 113(12.1) | <0.001 | 1312(84.2) | 253(15.8) | <0.001 |
| | 2 Quartile | 1334(95.9) | 59(4.1) | | 1847(92.7) | 155(7.3) | |
| | 3 Quartile | 1597(96.5) | 55(3.5) | | 2082(94.4) | 126(5.6) | |
| | 4 Quartile | 1682(97.4) | 41(2.6) | | 2204(96.4) | 72(3.6) | |
| Education level | ≤ Elementary school | 788(91.3) | 77(8.7) | <0.001 | 1705(85.1) | 289(14.9) | <0.001 |
| | Middle school | 580(94.9) | 34(5.1) | | 741(90.9) | 75(9.1) | |
| | High school | 1882(95.2) | 90(4.8) | | 2401(94.8) | 132(4.2) | |
| | ≥ University | 2203(96.9) | 67(3.1) | | 2598(92.9) | 110(7.1) | |

| | | Men | | Women | |
|---|---|---|---|---|---|
| | | **OR (95% CI)** | ***p*-Value** | **OR (95% CI)** | ***p*-Value** |
| Household Income | 1st | 5.082(3.283, 7.865) | <0.001 | 4.962(3.66, 6.726) | <0.001 |
| | 2nd | 1.569(0.991, 2.484) | 0.055 | 2.087(1.509, 2.887) | <0.001 |
| | 3rd | 1.34(0.843, 2.129) | 0.215 | 1.57(1.134, 2.174) | 0.007 |
| | 4th | ref | | ref | |
| Education level | ≤Elementary school | 3.007(2.023, 4.471) | <0.001 | 4.007(3.103, 5.175) | <0.001 |
| | Middle School | 1.688(1.013, 2.811) | 0.044 | 2.286(1.607, 3.251) | <0.001 |
| | High School | 1.604(1.1, 2.337) | 0.014 | 1.34(1.01, 1.778) | 0.042 |
| | ≥University | ref | | ref | |

**Supplementary Table S2. Relationship between lipid levels and household income & education levels in Men**

| Variables | Men *n*=5,721 | | | | | | | | | |
|---|---|---|---|---|---|---|---|---|---|---|
| | Household Income | | | | | Education Level | | | | |
| | 1 Quartile | 2 Quartile | 3 Quartile | 4 Quartile | *p*-Value | ≤ Elementary school | Middle School | High School | ≥ University | *p*-Value |
| | *n*=953 | *n*=1,393 | *n*=1,652 | *n*=1,723 | | *n*=865 | *n*=614 | *n*=1,972 | *n*=2,270 | |
| LDL cholesterol | | | | | <0.001 | | | | | <0.001 |
| <100 | 392(41.39) | 497(36.17) | 555(32.64) | 553(32.21) | | 365(42.2) | 250(38.63) | 761(39.58) | 621(27.6) | |
| 100-129 | 328(34.24) | 480(35.96) | 565(33.86) | 614(35.67) | | 293(32.17) | 212(34.68) | 659(33.87) | 823(36.61) | |
| 130-159 | 164(17.05) | 328(22.41) | 395(24.18) | 417(24) | | 158(20.36) | 125(20.97) | 407(19.36) | 614(26.62) | |
| 160-189 | 56(6.21) | 73(4.54) | 121(8.23) | 117(6.78) | | 39(4.31) | 22(4.48) | 128(6.45) | 178(7.67) | |
| ≥190 | 13(1.11) | 15(0.92) | 16(1.1) | 22(1.34) | | 10(0.96) | 5(1.24) | 17(0.74) | 34(1.51) | |
| Total cholesterol | | | | | 0.001 | | | | | <0.001 |
| <200 | 658(68.66) | 880(64.74) | 1007(59.61) | 1051(60.47) | | 613(69.47) | 417(64.65) | 1267(65.4) | 1299(57.53) | |
| 200-239 | 229(24.57) | 410(28.61) | 486(29.95) | 517(30.32) | | 205(24.82) | 165(28.22) | 532(26.2) | 740(32.61) | |
| ≥240 | 66(6.77) | 103(6.65) | 159(10.44) | 155(9.21) | | 47(5.7) | 32(7.13) | 173(8.4) | 231(9.85) | |
| HDL cholesterol | | | | | 0.013 | | | | | 0.014 |
| <40 | 287(28.78) | 380(26.42) | 415(23.83) | 403(22.18) | | 260(29.84) | 183(28.09) | 490(23.24) | 552(23.81) | |
| 40-59 | 559(61.24) | 838(61.44) | 1027(63.62) | 1088(63.88) | | 489(57.44) | 349(57.14) | 1227(64.25) | 1447(63.94) | |
| ≥60 | 107(9.98) | 175(12.14) | 210(12.55) | 232(13.94) | | 116(12.72) | 82(14.78) | 255(12.51) | 271(12.25) | |
| Triglyceride | | | | | 0.767 | | | | | 0.139 |
| <150 | 615(61.83) | 835(60) | 1010(60.58) | 1019(59.64) | | 550(60.85) | 390(60.19) | 1215(62.38) | 1324(58.39) | |
| 150-199 | 157(17.82) | 229(16.66) | 292(17.25) | 293(16.47) | | 144(17.88) | 88(14.95) | 325(15.9) | 414(17.95) | |
| ≥200 | 181(20.35) | 329(23.34) | 350(22.18) | 411(23.89) | | 171(21.27) | 136(24.86) | 432(21.72) | 532(23.66) | |

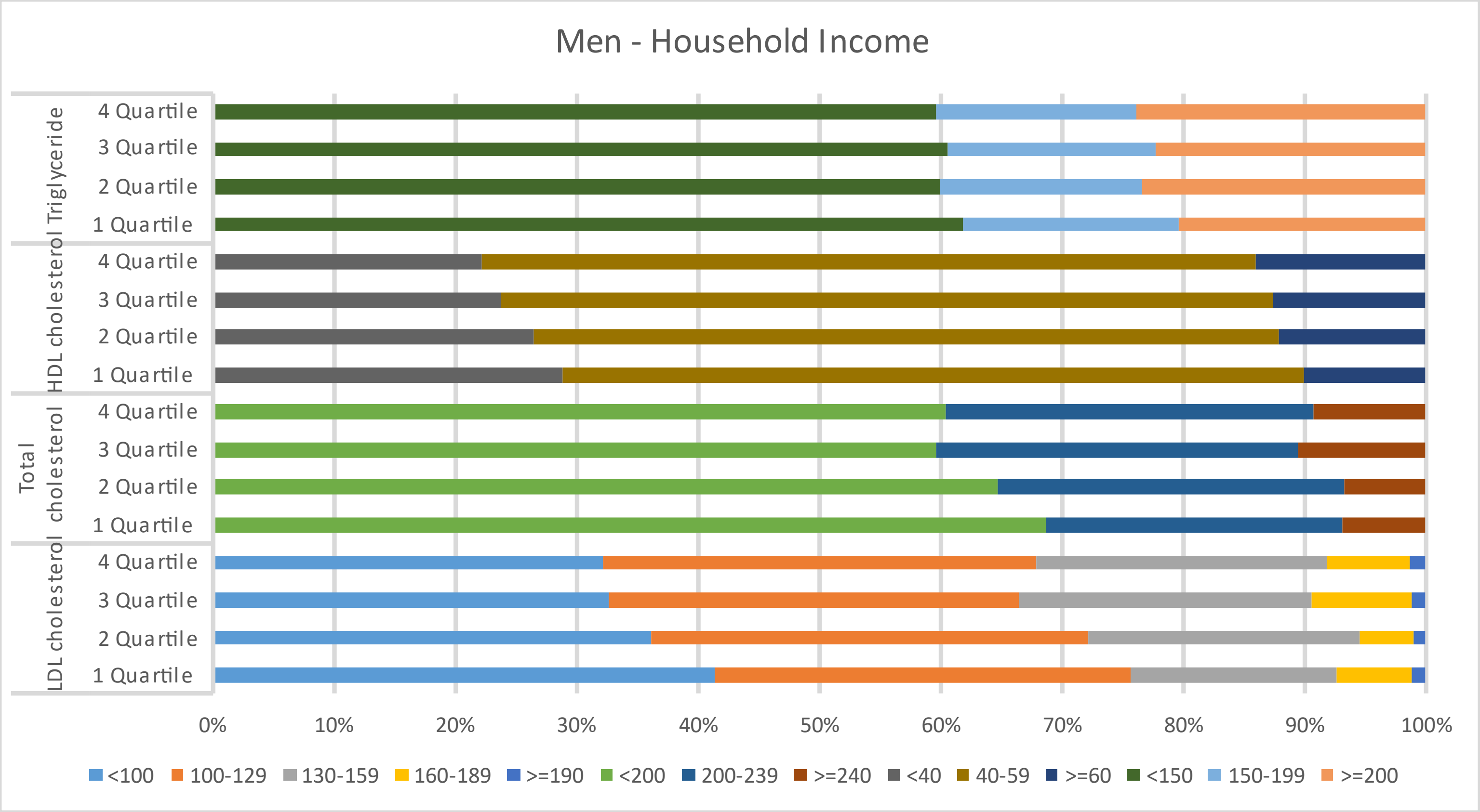

Men - Household Income
Triglyceride
HDL cholesterol
Total cholesterol
LDL cholesterol
4 Quartile
3 Quartile
2 Quartile
1 Quartile
0%
10%
20%
30%
40%
50%
60%
70%
80%
90%
100%
<100
100-129
130-159
160-189
>=190
<200
200-239
>=240
<40
40-59
>=60
<150
150-199
>=200

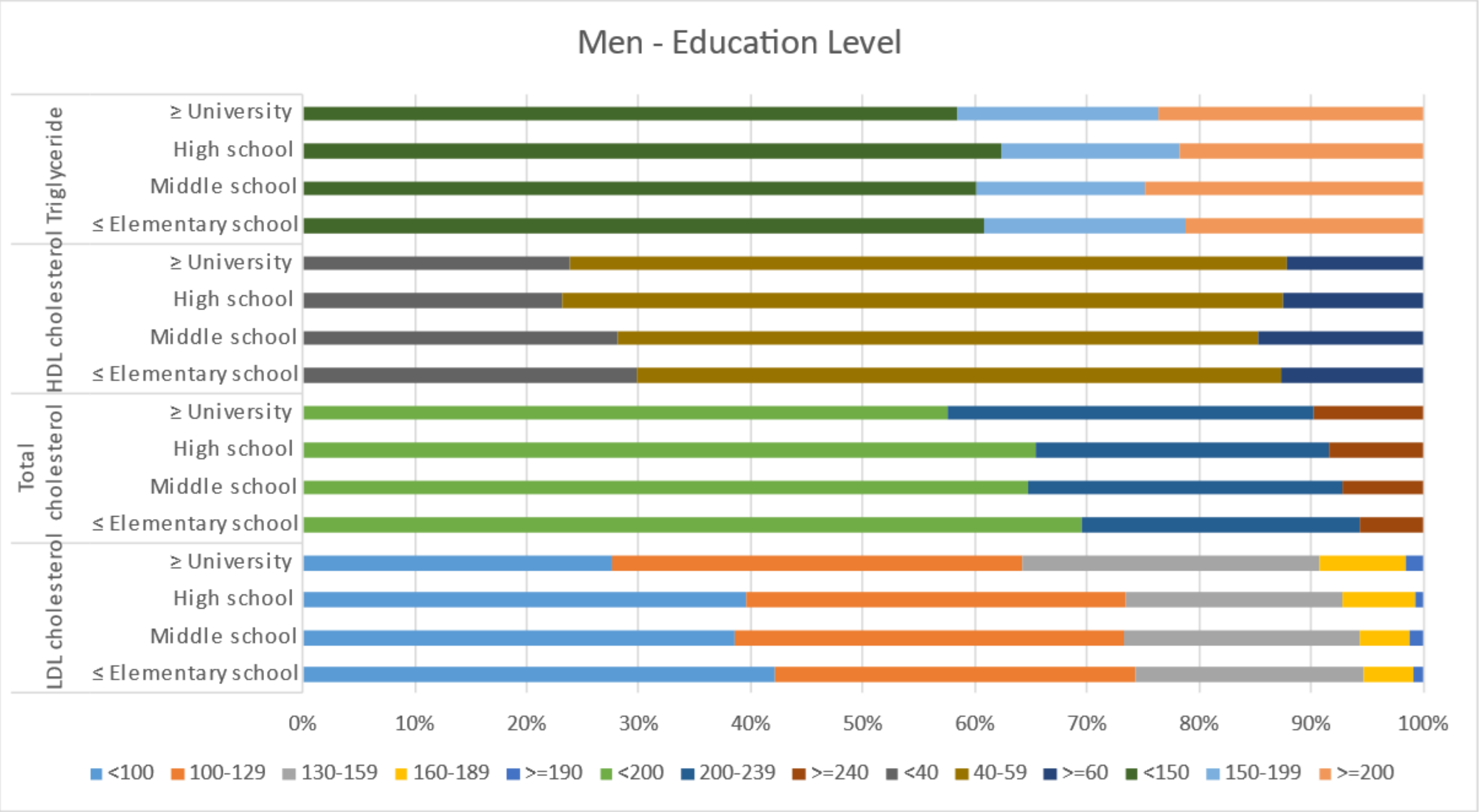

Men - Education Level
Triglyceride
HDL cholesterol
Total cholesterol
LDL cholesterol
≥ University
High school
Middle school
≤ Elementary school
0%
10%
20%
30%
40%
50%
60%
70%
80%
90%
100%
<100
100-129
130-159
160-189
>=190
<200
200-239
>=240
<40
40-59
>=60
<150
150-199
>=200

**Supplementary Table S3. Relationship between lipid levels and household income & education levels in Women**

| Variables | Women *n*=8,051 | | | | | | | | | |
|---|---|---|---|---|---|---|---|---|---|---|
| | **Household Income** | | | | | **Education level** | | | | |
| | **1 Quartile** *n*=1,565 | **2 Quartile** *n*=2,002 | **3 Quartile** *n*=2,208 | **4 Quartile** *n*=2,276 | ***p*-Value** | **≤ Elementary school** *n*=1,994 | **Middle School** *n*=816 | **High School** *n*=2,533 | **≥ University** *n*=2,708 | ***p*-Value** |
| LDL cholesterol | | | | | 0.231 | | | | | <0.001 |
| <100 | 548(34.72) | 671(35.27) | 702(31.63) | 744(33.59) | | 679(32.63) | 240(30.45) | 833(34.12) | 913(34.41) | |
| 100-129 | 508(34.85) | 731(36.71) | 850(39.29) | 890(39.82) | | 638(32.86) | 274(33.92) | 944(37.93) | 1123(41.94) | |
| 130-159 | 345(20.68) | 428(20.24) | 461(20.46) | 455(19.04) | | 455(23.12) | 200(23.82) | 540(19.97) | 494(17.55) | |
| 160-189 | 135(7.96) | 140(6.35) | 161(6.91) | 152(6.01) | | 182(8.88) | 84(9.97) | 174(6.34) | 148(5.05) | |
| ≥190 | 29(1.78) | 32(1.43) | 34(1.71) | 35(1.55) | | 40(2.51) | 18(1.84) | 42(1.64) | 30(1.06) | |
| Total cholesterol | | | | | 0.313 | | | | | <0.001 |
| <200 | 934(61.07) | 1210(62.75) | 1359(61.77) | 1415(63.62) | | 1178(57.64) | 428(54.96) | 1530(62.29) | 1782(66.89) | |
| 200-239 | 449(27.84) | 607(28.91) | 648(29.14) | 644(27.25) | | 582(30.02) | 287(32.67) | 765(28.81) | 714(25.86) | |
| ≥240 | 182(11.09) | 185(8.34) | 201(9.1) | 217(9.13) | | 234(12.34) | 101(12.37) | 238(8.9) | 212(7.24) | |
| HDL cholesterol | | | | | <0.001 | | | | | <0.001 |
| <40 | 283(16.28) | 226(10.2) | 186(8.25) | 182(7.18) | | 375(18.19) | 117(14.58) | 231(8.91) | 154(4.99) | |
| 40-59 | 942(59.95) | 1202(59.17) | 1346(59.7) | 1265(54.32) | | 1265(62.84) | 496(60.49) | 1494(57.96) | 1500(54.93) | |
| ≥60 | 340(23.77) | 574(30.63) | 676(32.05) | 829(38.5) | | 354(18.98) | 203(24.94) | 808(33.14) | 1054(40.08) | |
| Triglyceride | | | | | <0.001 | | | | | <0.001 |
| <150 | 1087(71.29) | 1548(79.8) | 1767(80.94) | 1889(83.74) | | 1332(66.81) | 595(72.56) | 2017(80.82) | 2347(87.55) | |
| 150-199 | 244(14.35) | 230(9.8) | 253(10.58) | 202(8.68) | | 344(16.91) | 112(13.12) | 268(9.86) | 205(7.03) | |
| ≥200 | 234(14.36) | 224(10.4) | 188(8.48) | 185(7.58) | | 318(16.27) | 109(14.32) | 248(9.32) | 156(5.42) | |

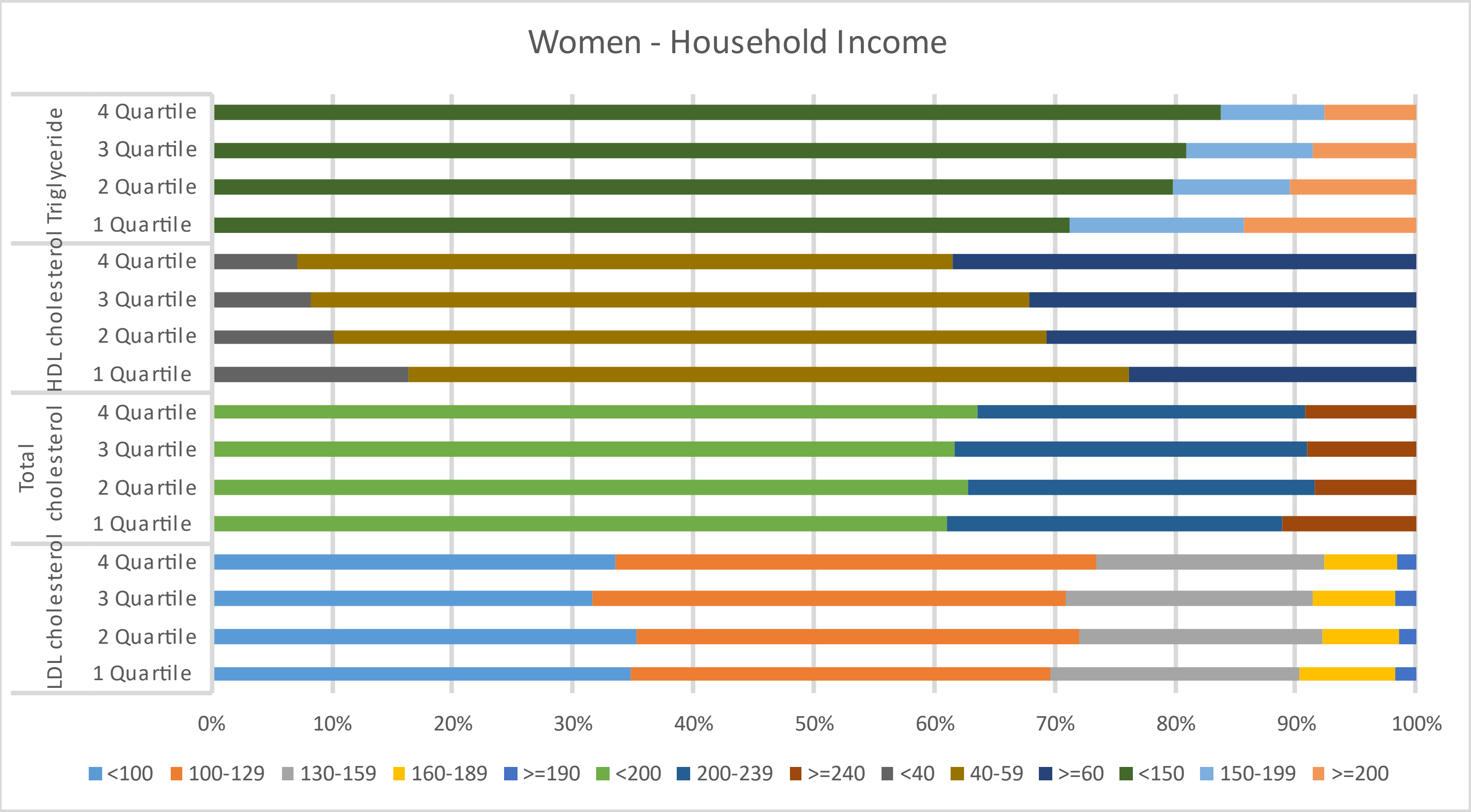

Women - Household Income
Triglyceride
HDL cholesterol
Total cholesterol
LDL cholesterol
4 Quartile
3 Quartile
2 Quartile
1 Quartile
0%
10%
20%
30%
40%
50%
60%
70%
80%
90%
100%
<100
100-129
130-159
160-189
>=190
<200
200-239
>=240
<40
40-59
>=60
<150
150-199
>=200

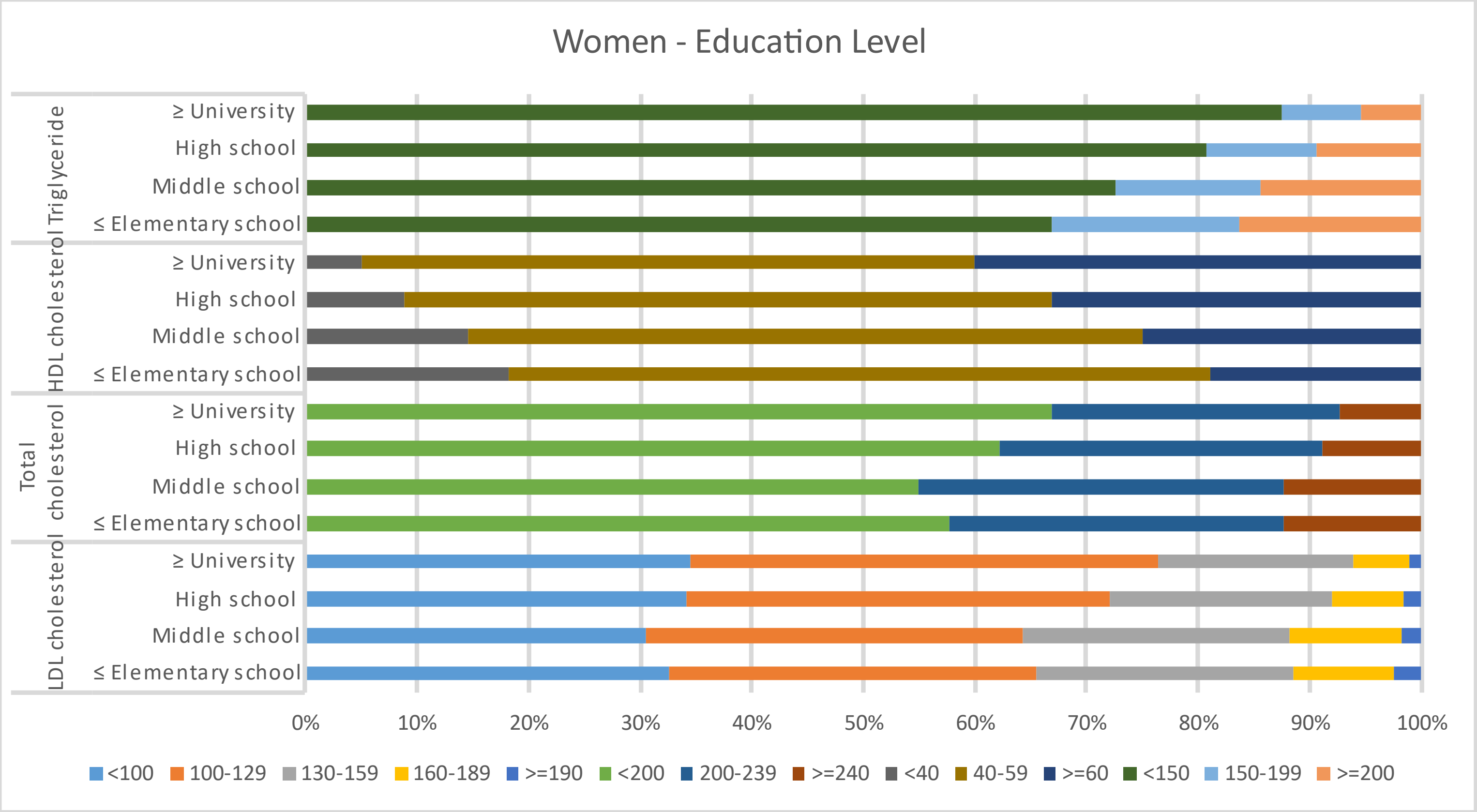
Women - Education Level
Triglyceride
HDL cholesterol
Total cholesterol
LDL cholesterol
≥ University
High school
Middle school
≤ Elementary school
0%
10%
20%
30%
40%
50%
60%
70%
80%
90%
100%
<100
100-129
130-159
160-189
>=190
<200
200-239
>=240
<40
40-59
>=60
<150
150-199
>=200
